# Medical School Experiences Associated with Change in Implicit Racial Bias Among 3547 Students: A Medical Student CHANGES Study Report

**DOI:** 10.1007/s11606-015-3447-7

**Published:** 2015-07-01

**Authors:** Michelle van Ryn, Rachel Hardeman, Sean M. Phelan, Diana J. Burgess PhD, John F. Dovidio, Jeph Herrin, Sara E. Burke, David B. Nelson, Sylvia Perry, Mark Yeazel, Julia M. Przedworski

**Affiliations:** Mayo Clinic College of Medicine, Rochester, MN USA; University of Minnesota, Minneapolis, MN USA; Center for Chronic Disease Outcomes Research, Minneapolis Veterans Affairs Medical Center, Minneapolis, MN USA; Yale University, New Haven, CT USA; University of Vermont, Burlington, VT USA

**Keywords:** disparities, medical education, implicit racial bias, physician–patient relations, attitude of health personnel

## Abstract

**BACKGROUND:**

Physician implicit (unconscious, automatic) bias has been shown to contribute to racial disparities in medical care. The impact of medical education on implicit racial bias is unknown.

**OBJECTIVE:**

To examine the association between change in student implicit racial bias towards African Americans and student reports on their experiences with 1) formal curricula related to disparities in health and health care, cultural competence, and/or minority health; 2) informal curricula including racial climate and role model behavior; and 3) the amount and favorability of interracial contact during school.

**DESIGN:**

Prospective observational study involving Web-based questionnaires administered during first (2010) and last (2014) semesters of medical school.

**PARTICIPANTS:**

A total of 3547 students from a stratified random sample of 49 U.S. medical schools.

**MAIN OUTCOME(S) AND MEASURE(S):**

Change in implicit racial attitudes as assessed by the Black-White Implicit Association Test administered during the first semester and again during the last semester of medical school.

**KEY RESULTS:**

In multivariable modeling, having completed the Black-White Implicit Association Test during medical school remained a statistically significant predictor of decreased implicit racial bias (−5.34, p ≤ 0.001: mixed effects regression with random intercept across schools). Students' self-assessed skills regarding providing care to African American patients had a borderline association with decreased implicit racial bias (−2.18, *p* = 0.056). Having heard negative comments from attending physicians or residents about African American patients (3.17, *p* = 0.026) and having had unfavorable vs. very favorable contact with African American physicians (18.79, *p* = 0.003) were statistically significant predictors of increased implicit racial bias.

**CONCLUSIONS:**

Medical school experiences in all three domains were independently associated with change in student implicit racial attitudes. These findings are notable given that even small differences in implicit racial attitudes have been shown to affect behavior and that implicit attitudes are developed over a long period of repeated exposure and are difficult to change.

## INTRODUCTION

Over the past two decades, hundreds of studies have documented widespread racial inequalities in medical care.^1^,^2^ Disparities in health and health care relative to white populations have been most extensively and consistently documented for African Americans, but also occur for other racial and ethnic groups in the U.S. While the causes of unequal care for African Americans are complex, provider behavior and decision-making is one documented contributor.^1^–^7^ Explanations for the provision of unequal care by physicians draw heavily from research on *implicit* racial bias.^7^–^9^ This term refers to automatic and unconscious negative attitudes towards African Americans as compared to whites. Implicit racial bias influences behavior in unintentional but powerful and systematic ways,^10^ profoundly influencing clinical decision-making.^11^,^12^ In addition, implicit racial bias predicts nonverbal behavior such as eye contact and posture,^13^–^17^ and has been shown to influence the quality of physicians’ interpersonal communication with African American patients and, in turn, patients’ trust and perceptions of their physicians.^4^,^18^,^19^

Over a decade ago, the Institute of Medicine called for investigation into the way socialization into the “culture of medicine” promotes or inhibits physician expressions of racial bias.^20^ In response to this, and to the continued evidence of physician bias, many medical schools have developed curricula aimed at eliminating physician contribution to inequality in care, although little is known about their effectiveness in reducing implicit racial bias. Additionally, the effect of the informal or “hidden” curricula (informal climate and role model behavior) on racial attitudes of medical student is unknown.^21^–^25^ Thus, medical educators have limited information to inform best strategies to address implicit racial bias during training in medical school.

The Medical Student Cognitive Habits and Growth Evaluation Study (CHANGES) sought to address this gap in evidence using a longitudinal multi-measure design with a large sample of students attending a stratified random sample of 49 U.S. allopathic medical schools. Here we report on tests of our hypothesis that medical school exposure in three domains would predict change in non-African American medical student implicit racial bias towards African Americans. The three domains were chosen based on a significant body of evidence indicating their potential combined and independent impact on learner attitudes and outcomes and/or on inter-racial interactions.^15^,^22^–^40^

1. **Formal curricula** refers to what is explicitly taught in planned educational experiences and includes both information and skill-building.^26^ Student experiences in this domain include participation in planned educational activities targeting quality of care for African American (and other minority) patients, cultural competence, and/or interpersonal quality of care generally.
2. **Informal curricula,** sometimes described as the “hidden” curricula, is a term used to describe the sources of lessons absorbed outside of formal curricula.^27^ Informal curricula conveys information on how to interact with patients, how to perceive and respond to individuals from different social groups, and general rules for acceptable behavior. The definition of informal curriculum corresponds to definitions of *informal organizational culture*, a powerful determinant of behaviors and attitudes of organizational members.^34^,^35^ Educational research and theory has indicated that informal curricula is largely delivered through the behavior of faculty (role models) and organizational cultural and climate.^33^
3. The amount and favorability of **interracial contact** has been shown in a large body of evidence to influence interracial attitudes and behavior.^15^,^36^–^41^ Experiences in this domain include interactions with African American clerical staff, allied health staff, medical students, and physicians.

## METHODS

### Overview

This was a prospective observational study in which medical students completed questionnaires and measures of implicit racial bias during their first and fourth years of medical school. We examined the association between medical school experiences and changes in implicit racial bias (IRBIAS) between the first and fourth years among non-African American students using mixed effects regression models.

### Study Sample

**Fig. 1:**
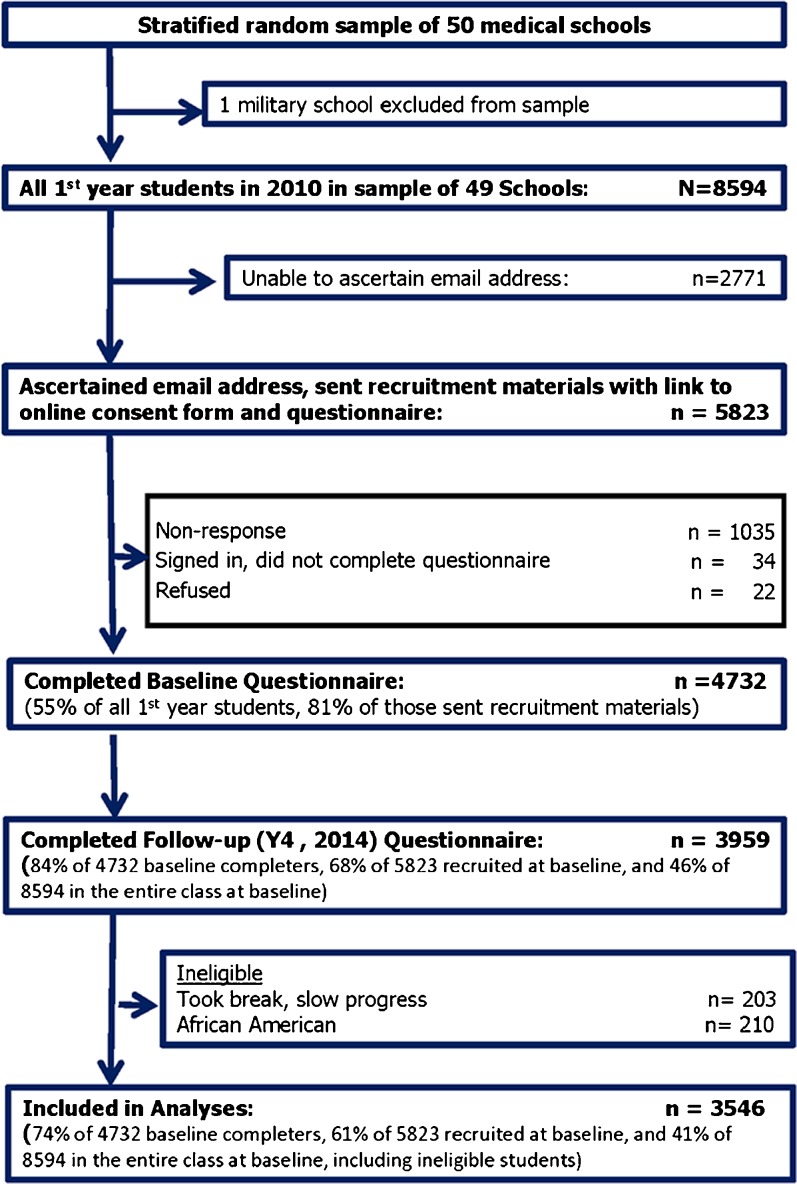
Study sample flow chart

CHANGES is a longitudinal study of students who matriculated to 49 U.S. medical schools in the fall of 2010. Schools were randomly selected using a sample proportional to strata size methodology (see van Ryn et al.^42^ for descriptions of baseline ascertainment and recruitment strategy). Figure 1 provides a detailed sample flow chart. In the fall of 2010 we invited 5823 first year students to complete the baseline Web-based survey. Of these, 4732 (81 %) responded. In spring 2014, we invited the 4732 baseline responders to complete the follow-up measures, and 3959 (84 %) responded. Students who had taken a break or had slow progress through school for any reason were excluded. As the study was designed to test hypotheses specific to attitudes among non-African American students, African American students were excluded, leaving 3546 eligible for analysis.

### Measures

Standard survey questions were used to measure demographic characteristics. Students’ family household income during their time in high school was used to estimate family-of-origin socioeconomic status.

Measures assessing student exposure to **formal curricula** are shown in Table 1. Since coursework and experiences vary among students within a school, we assessed student reports on their training experiences rather than interviewing school personnel. All are single items, with the exception of *self-efficacy (self-assessed skills) regarding providing care to African American patients*, which was estimated with a three-item scale. Self-efficacy can be influenced by many factors. It was included in the formal curricula domain because it is a frequently used measure of the impact of formal curricula.^15^,^43^–^45^ Students reported on the degree to which they felt: ‘prepared to handle a patient who is a member of a racial or ethnic minority’; ‘skilled at overcoming unintended or unconscious racial bias’; and ‘skilled in developing a positive relationship with racial minority patients’. **Informal curricula** were assessed in four ways. The first two assessments came from two *Racial Climate Scale*^46^ subscales: 1) *Racial Tension*, which includes items like ‘The interracial climate on this campus is tense’, and 2) *Medical School Effort*, which includes items like ‘This medical school… makes a genuine effort to recruit racial and ethnic minority students’. 3) The degree to which the school had a *Learning Orientation to Racial Relations*^47^ was assessed by a two-item scale. Items included ‘Students in this medical school… have the opportunity to learn how to interact more effectively with members of another race’ and ‘…are encouraged to learn from their mistakes in interacting with members of another race.’ 4) *Faculty/Role Model Behavior* was assessed by asking how often respondents ‘witnessed attending or resident physicians making negative or derogatory remarks about black patients’. The amount and favorability of **interracial contact** was measured by items assessing, separately, the amount and favorability of contact they had with “black” medical students, faculty, attending physicians and/or residents, allied health staff, and clerical and administrative staff.

**Table 1:** Distribution of Responses on Medical School Experiences (*n* = 3547)

Formal Curricula			
	Percentage that answered ‘Yes’	Number	
Taken a seminar on minority health	52.3 %	1856	
Participated in a cultural awareness course/workshop	75.7 %	2686	
Completed an implicit association test (IAT) of unconscious racial bias	24.5 %	869	
	Mean (SD)	Minimum	Maximum
Hours of training devoted to communication skills	28.97 (16.56)	0	50
Hours of training devoted to partnership-building skills	18.29 (15.32)	0	50
Hours of training devoted to racial disparities in health care	13.85 (11.82)	0	50
Hours of training devoted to identifying cultural customs that might affect clinical care	13.49 (11.57)	0	50
Hours of training devoted to the potential effect of unintended racial bias on the care you provide	8.81 (10.23)	0	50
Hours of training devoted to seeing things from your patient’s perspective	21.08 (15.40)	0	50
How prepared are you to handle a patient from a culture different from your own	4.09 (.78)	1	5
Self-efficacy regarding providing care for black patients (scale)	3.69 (.67)	1	5
Informal Curricula			
	Percentage that answered ‘Yes’	Number	
Ever heard residents or attendings make negative comments about black patients	48.7 %	1728	
	Mean (SD)	Minimum	Maximum
Learning Orientation to Racial Relations Scale	5.39 (.50)	1	7
Racial Climate Scale: Racial Tension Subscale	3.17 (1.44)	1	7
Racial Climate Scale: Medical School Effort Subscale	5.61 (1.17)	1	7
Interracial Contact			
	Percentage	Number	
How much interaction with black medical students			
1 None	3.0	107	
2 Little	26.1	925	
3 Some	39.0	1385	
4 Substantial	29.9	1059	
Favorability of interaction with black medical students			
1 Very unfavorable	.6	23	
2 Unfavorable	1.8	65	
3 Favorable	41.8	1481	
4 Very favorable	53.0	1880	
How much interaction with black faculty, attending physicians and residents			
1 None	2.6	91	
2 Little	32.6	1158	
3 Some	43.6	1545	
4 Substantial	19.2	682	
Favorability of interaction with black faculty, attending physicians and residents			
1 Very unfavorable	.5	17	
2 Unfavorable	1.4	49	
3 Favorable	42.9	1520	
4 Very favorable	52.4	1860	
How much interaction with black allied health staff			
1 None	3.7	133	
2 Little	17.1	606	
3 Some	35.7	1266	
4 Substantial	41.4	1468	
Favorability of interaction with black allied health staff			
1 Very unfavorable	1.0	37	
2 Unfavorable	4.9	174	
3 Favorable	50.4	1787	
4 Very favorable	40.3	1429	
How much Interaction with black clerical, administrative and secretarial staff			
1 None	2.3	83	
2 Little	15.0	532	
3 Some	33.2	1178	
4 Substantial	47.4	1682	
Favorability of interaction with black clerical, administrative and secretarial staff			
1 Very unfavorable	1.5	54	
2 Unfavorable	7.2	256	
3 Favorable	48.4	1715	
4 Very favorable	39.9	1415	

#### Independent Sample Validation of Racial Climate Scores

We administered the three racial climate measures (*Racial Tension*, *Medical School Effort*, and *Learning Orientation To Racial Relations*) to an independent sample of students (*n* = 1778) in their fourth year of training in the same 49 schools while the study cohort was in their third year (2013). Correspondence between the independent sample and the study sample estimates were high (*Racial Tension*r = 0.87, *Medical School Effort* = 0.84, *Learning Orientation to Racial Relations*r = 0.95), suggesting that scores on these measures reflect an underlying aspect of the school racial climate vs. individual or cohort characteristics.

Implicit racial bias (IRBIAS) against African Americans was assessed using the Black-White Implicit Association Test (IAT)^48^–^50^ during students’ first (Y1) and last (Y4) semesters of medical school. The Black-White IAT uses reaction times to assess the strength of *automatic* associations between race (black, white) and evaluations (e.g., good, bad), and is very difficult to counterfeit.^51^–^56^ The IAT has consistently and significantly predicted a wide range of judgments, choices, physiological responses, and behaviors,^57^ and has been shown to be a better predictor of discrimination toward a racial group than attitudes measured by self-report.^15^,^57^–^60^ More information about IAT properties and administration can be found at https://implicit.harvard.edu/implicit/iatdetails.html. IAT scores were multiplied by 100 for ease of interpretation. The dependent variable for this study, change in IRBIAS, was computed using a simple difference score (Y4 IAT score − Y1 IAT score). Higher scores represent increases in IRBIAS against African Americans.

### Analyses

Descriptive statistics were used to characterize the sample and to explore the bivariate associations between sociodemographic characteristics of the cohort and change in IRBIAS. Since student responses and outcomes likely correlated within medical schools, we used methods appropriate for clustered data in all analyses. We examined the bivariate association for the items in each of the three domains (formal curricula, informal curricula, and interracial contact) using mixed effects models with a random intercept across schools to test for an association. We then examined the relationship between the overall school mean for each of the independent variables and change in IRBIAS. We used a set of sequential analyses to reduce survey items to a more parsimonious set that would be most strongly associated with change in IRBIAS. Those that were associated (*p* < 0.10) were retained, and variance decomposition was used to identify collinear predictors within each domain; when two or more variables had variance decomposition portions greater than 50 %, we retained the one with the greatest percentage of variation explained in the bivariate analysis.^61^ The retained items were used as independent variables in three separate models—one model for each domain; those that were significant predictors independent of the other variables within their domain (*p* < 0.10) were then carried to a final model. This approach has been used previously in health research to reduce a large number of related factors to a more parsimonious set.^62^,^63^ This final model was then expanded to include student characteristics. Baseline (Y1) IAT scores were included in all models in order to account for baseline IRBIAS assessed upon entry to medical school.

All study procedures were reviewed and approved by the institutional review boards at the Mayo Clinic, University of Minnesota, and Yale University. All analysis was performed in Stata version 13.1 (released 2014; StataCorp LP, College Station TX, USA) and SPSS version 22.0 (released 2013; IBM Corp., Armonk, NY, USA).

## RESULTS

At the year 4 follow-up, the average age of respondents was 27.8 years (SD 2.49, range 23–53). Half were women (49.3 %). Family-of-origin annual income levels ranged from less than $30,000 (4.7 %) to $250,000 or more (18.8 %), with around one-third reporting a family income of $100,000–250,000 (36.1 %). Hispanic students comprised 5.6 % and Asian students 24.7 % of the sample, consistent with the race/ethnicity distribution reported by the American Association of Medical Colleges for all medical students.^64^ There were no statistically significant relationships between student sociodemographic factors and change in IRBIAS.

The distribution of responses on each item within the three domains is provided in Table 1. The bivariate relationship between school factors and change in IRBIAS, adjusted for baseline IRBIAS, is provided in Table 2. Negative coefficients represent a decrease in IRBIAS. Most variables within the **formal curricula** domain had a statistically significant bivariate association with a decrease in IRBIAS. Within the **informal curricula** domain, negative role modeling was associated with an increase, and learning orientation with a decrease, in IRBIAS. Within the **interracial contact** domain, unfavorable contact with African American faculty, medical students, allied health and administrative staff were all associated with increases in IRBIAS, as was a lower amount of contact with African American medical students and faculty. Interactions between amount and favorability of contact did not have a significant bivariate relationship with change in IRBIAS. All coefficients represent small effect sizes.

**Table 2:** Bivariate Relationship Between Medical School Experiences and Change in Student Implicit Racial Bias

	Coefficient (SE)	*p* value
Domain 1: FORMAL CURRICULA		
Took a seminar (course, workshop) on minority health (ref = No)	−3.9 (1.4)	0.006
Took a seminar (course, workshop) on cultural awareness or competence (ref = No)	−3.7 (1.7)	0.028
Completed a Race Implicit Association Test as part of medical school (ref = No)	−5.7 (1.6)	0.001
Hours of training devoted to racial disparities in health care	−0.2 (0.1)	0.002
Hours of training devoted to identifying cultural customs that might affect care	−0.1 (0.1)	0.031
Hours of training devoted to seeing things from patient’s perspective (cognitive empathy)	−0.1 (0.0)	0.053
Hours of training devoted to communication skills	−0.1 (0.0)	0.128
Self-efficacy regarding providing care for black patients (scale)	−3.2 (1.1)	0.002
Domain 2: INFORMAL CURRICULA		
Heard attendings or residents make negative comments about black patients *(ref = No)*	3.9 (1.4)	0.006
Learning Orientation to Interracial Interactions Scale	−1.5 (0.5)	0.006
Racial Climate Scale: Racial Tension Subscale	0.6 (0.5)	0.196
Racial Climate Scale: Medical School Effort Subscale	−0.7 (0.6)	0.248
Domain 3: INTERRACIAL CONTACT		
Amount of interaction with black medical students *(ref = Substantial)*		0.021
None	11.8 (4.3)	
Little	3.7 (1.9)	
Some	1.4 (1.7)	
Favorability of interaction with black medical students *(ref = Very favorable)*		0.007
Very unfavorable	6.8 (8.7)	
Unfavorable	12.3 (5.3)	
Favorable	4.0 (1.4)	
Amount of interaction with black faculty, attending physicians and/or residents *(ref = Substantial)*		0.03
None	9.2 (4.6)	
Little	5.2 (2.0)	
Some	4.6 (1.9)	
Favorability of interaction with black faculty, attending physicians and/or residents *(ref = Very favorable)*		<0.001
Very unfavorable	16.3 (10.1)	
Unfavorable	21.3 (6.2)	
Favorable	3.0 (1.4)	
Amount of interaction with black allied health staff *(ref = Substantial)*		0.315
None	4.3 (3.8)	
Little	3.1 (2.0)	
Some	0.2 (1.6)	
Favorability of interaction with black allied health staff *(ref = Very favorable)*		0.002
Very unfavorable	21.3 (6.7)	
Unfavorable	6.7 (3.3)	
Favorable	2.7 (1.5)	
Amount of interaction with black clerical and administrative staff *(ref = Substantial)*		0.65
None	1.2 (4.8)	
Little	2.5 (2.1)	
Some	0.1 (1.6)	
Favorability of interaction with black clerical and administrative staff *(ref = Very Favorable)*		<0.001
Very unfavorable	13.1 (5.7)	
Unfavorable	10.9 (2.8)	
Favorable	2.7 (1.5)	

Mixed effects models with random intercept across schools; adjusted for year 1 implicit racial bias. Positive coefficients indicate an increase in implicit racial bias

Table 3 includes the variables that had statistically significant bivariate associations with change in IRBIAS, and shows the results of estimates of this relationship, while adjusting for other variables *within the same domain* and baseline IRBIAS. Of the **formal curricula** variables, having taken an IAT as part of medical school training and self-efficacy regarding care for African American patients persisted in their statistically significant association with reductions in IRBIAS. Of the **informal curricula** variables, negative role modeling had a statistically significant association with increases, and school learning orientation with decreases, in IRBIAS. Of the **interracial contact** variables, unfavorable contact with African American faculty and African American allied health staff had borderline statistically significant associations with increased IRBIAS. In the same results described differently, highly favorable contact with African American faculty and allied health staff were associated with decreases in IRBIAS. Effect sizes remained small.

**Table 3:** Multivariable Relationship Between Medical School Experiences and Change in Student Implicit Racial Bias

Model 1: Multivariable Relationship Between Participation in Formal Curricula and Change in Student Implicit Racial Bias^*^			
Training	Coefficient (SE)	Statistical Significance	
Took a seminar (course, workshop) on minority health (ref = No)	−2.15 (1.12, −5.41)	0.198	
Took a seminar (course, workshop) on cultural awareness or competence (ref = No)	1.74 (−2.13, 5.61)	0.377	
Completed Race Implicit Associations Test as part of medical school (ref = No)	−4.49 (−1.12, −7.86)	0.009	
Hours of training devoted to racial disparities in health care	−0.15 (−0.33, 0.04)	0.114	
Hours of training devoted to identifying cultural customs that might affect care.	0.05 (−0.15, 0.24)	0.633	
Hours of training devoted to seeing things from patient’s perspective (cognitive empathy)	−0.05 (−0.16, 0.06)	0.392	
Self-efficacy regarding providing care for black patients (scale)	−2.38 (−4.62, −0.14)	0.037	
Model 2: Multivariable Relationship Between Medical School Informal Curricula and Change in Student Implicit Racial Bias^1^			
Climate	Coef. (SE)	*P* Value	
Heard attendings or residents make negative comments about black patients *(ref = No)*	3.66 (0.90, 6.43)	0.009	
Learning Orientation to Interracial Interactions Scale	−1.36 (−2.40, −0.32)	0.01	
Model 3: Multivariable Relationship Between Interracial Contact and Change in Student Implicit Racial Bias^1^			
Interracial Contact	Coefficient (SE)	Statistical Significance	Wald P
Amount of interaction with black medical students *(ref = Substantial)*			0.180
None	8.75 (−0.48, 17.99)	0.063	
Little	1.60 (−3.18, 6.37)	0.512	
Some	−0.76 (−4.65, 3.13)	0.701	
Amount of interaction with black faculty, attending physicians and/or residents *(ref = Substantial)*			0.233
None	7.30 (−3.16, 17.75)	0.171	
Little	3.40 (−1.70, 8.50)	0.191	
Some	4.21 (−0.11, 8.53)	0.056	
Favorability of interaction with black faculty, attending physicians and/or residents (*ref = Very Favorable*)			0.057
Very unfavorable	3.17 (−18.62, 24.96)	0.776	
Unfavorable	17.76 (5.04, 30.49)	0.006	
Favorable	1.39 (−2.61, 5.40)	0.496	
Favorability of interaction with black allied health staff (*ref = Very favorable*)			0.083
Very unfavorable	18.10 (3.52, 32.69)	0.015	
Unfavorable	3.84 (−3.24, 10.91)	0.288	
Favorable	0.83 (−3.22, 4.88)	0.687	

*Adjusted for other factors within the same domain and Y1 implicit bias. Mixed effects models with random intercept across schools. Positive coefficients indicate an increase in implicit racial bias

In the final multivariate model (Table 4), having completed the Black-White Implicit Association Test during medical school remained a statistically significant predictor of decreased IRBIAS (−5.34, p ≤ 0.001: mixed effects regression with random intercept across schools). Student self-efficacy regarding providing care to African American patients had a borderline association with decreased IRBIAS (−2.18., *p* = 0.056). Having heard attending physicians’ or residents’ make negative comments about African American patients, (3.17, *p* = 0.026) and having had unfavorable vs. very favorable contact with African American physicians (18.79, *P* = 0.003) remained statistically significant predictors of increased IRBIAS.

**Table 4:** Multivariable Relationship Between Statistically Significant Factors in all Domains and Change in Medical Student Implicit Racial Bias

	Coefficient (SE)	Statistical Significance	Wald p
Domain 1: FORMAL CURRICULA			
Completed a race implicit association test as part of medical school (ref = No)	−5.34 (−2.15, −8.53)	0.001	
Adequacy of training: Self-efficacy regarding working effectively with black patients (scale)	−2.18 (−4.42, 0.06)	0.056	
Domain 2: INFORMAL CURRICULA			
Heard attendings or residents make negative comments about black patients (ref = No)	3.17 (0.39, 5.96)	0.026	
Learning Orientation to Interracial Interactions Scale	−0.67 (−1.81, 0.46)	0.246	
Domain 3: INTERRACIAL CONTACT			
Favorability of interaction with black faculty, attending physicians and/or residents (ref = Very favorable)			0.019
Very unfavorable	7.71 (−12.83, 28.25)	0.462	
Unfavorable	18.79 (6.61, 30.98)	0.003	
Favorable	1.63 (−1.30, 4.57)	0.275	

Mixed effects models with random intercept across schools.; adjusted for year 1 implicit racial bias. Positive coefficients indicate an increase in implicit racial bias

## DISCUSSION

In this cohort of 3764 medical students, one or more medical school experiences within each of the three domains, (formal curricula, informal curricula, and interracial contact) had small but statistically significant associations with changes in IRBIAS. These findings are important, as very small differences in implicit racial attitudes have been shown to affect behavior.^65^ Implicit attitudes are developed over a long period of repeated exposure, consistently reinforced by cultural factors, and are notoriously difficult to change.^10^,^66^–^69^

### Formal Curricula

Many of the training experiences within this domain had small but significant bivariate associations with change in IRBIAS. However, when adjusted for each other and baseline implicit bias (Table 3), having completed an IAT as part of medical training and self-efficacy regarding care for African American patients remained the only statistically significant predictors of a decrease in IRBIAS. These findings warrant further investigation. The impact of taking an IAT may be reflected by an increase in the relevance of and perceived need for bias reduction. Alternately, if instructors who included an IAT in their classes were systematically different from those who did not, what appears to be an association between the IAT and change in IRBIAS might be spurious, reflecting instead an association between a quality of the instructors and reduction in student IRBIAS. For example, it is possible that instructors who included an IAT had a deeper understanding or comfort with discussing IRBIAS. In either case, the finding supports the benefit of implementing curricula taught by instructors with sufficient depth of knowledge of the nature of IRBIAS to effectively and appropriately incorporate implicit self-assessment into their curriculum.

Student self-efficacy regarding providing care to African American patients remained statistically significant in the model, adjusting for other training variables and baseline IAT. It is possible that the bivariate associations between the training variables and change in implicit bias (Table 2) were mediated by the impact of the training experiences on student self-efficacy regarding care for African American patients. Nevertheless, these findings support the recommendation that medical schools evaluate their current training, and implement and evaluate improved training, intended to increase non-African American students’ self-efficacy in providing care for African American patients.

### Informal Curricula

The degree to which the school had a learning orientation to interracial interactions and students’ exposure to negative role modeling in the form of negative comments from faculty and attendings were associated with a change in implicit bias in bivariate analyses (Table 2), and remained statistically significant after adjusting for each other and Y1 IRBIAS (Table 3); learning orientation was associated with a greater decrease, and exposure to negative role modelling with a greater increase, in IRBIAS. When formal training and interracial contact were entered into the model (Table 4), learning orientation dropped from the model while negative role modelling persisted in having a statistically significant detrimental effect on IRBIAS, reinforcing the assertion that medical schools have powerful informal or “hidden” curricula that can be “unearthed in the language used”.^25^ These findings point to the need for medical schools, in partnership with clinical training sites, to assess, monitor and, if needed, intervene to improve racial attitudes and behaviors among attending and resident physicians.

### Interracial Contact

Students who reported having had highly favorable contact with African American faculty had decreased racial bias, while those who reported unfavorable contact had increased racial bias. However, only 46 students reported having unfavorable, and 17 very unfavorable, contact with African American faculty, residents and attending physicians, making these results difficult to interpret. Student reports on the amount of contact with African American faculty, residents and attending physicians were not associated with change in IRBIAS, but this may reflect very limited opportunities for contact. African Americans make up only around 3 % of medical school faculty,^70^ and in that group, a significant number are practicing physicians with very limited or non-existent teaching roles.^71^ These findings add support to long-standing recommendations to increase the number of African American physicians in faculty roles, especially those that provide opportunities for positive interactions with medical students.^2^,^72^–^75^

This study had several limitations. First, findings may not generalize to all medical students. Second, combining faculty and resident physicians in a single category may have obscured any impact of one or the other group. Third, interpretation of the terms contained in the measures (e.g., “unintended or unconscious racial bias”) may have been affected by whether these concepts were included in their curricula. Fourth, measures of the formal curricula only assessed exposure to topics—the actual quality of the formal curricula is unknown. Fifth, self-reported data on school experiences may have been influenced by student characteristics, and students may have differed in how they interpreted response options. While this last limitation is common to all studies involving self-reporting, the independent sample validation of the racial climate measures and scores was reassuring.

The results of this study indicate that while curricula is important, a sole focus on improving curricula will be insufficient for addressing medical school contributors to graduating student implicit racial bias. The deleterious impact of negative role model behavior speaks to the importance of overall culture change in medical schools and clinical training sites. Findings that medical school experiences were associated with changes in student implicit racial bias point to the potential for medical education to reduce physician contribution to racial disparities in health care.
